# Nerve enlargement differs among chronic inflammatory demyelinating polyradiculoneuropathy subtypes and multifocal motor neuropathy

**DOI:** 10.1016/j.cnp.2023.10.002

**Published:** 2023-11-22

**Authors:** Masaaki Yoshikawa, Kenji Sekiguchi, Hirotomo Suehiro, Shunsuke Watanabe, Yoshikatsu Noda, Hideo Hara, Riki Matsumoto

**Affiliations:** a Division of Neurology, Kobe University Graduate School of Medicine, 7-5-1 Kusunoki-cho, Chuo-ku, Kobe, Hyogo 650-0017, Japan; b Division of Neurology, Department of Internal Medicine, Saga University Faculty of Medicine, 5-1-1 Nabeshima-cho, Saga 849-8501, Japan; c Fukuoka International University of Health and Welfare, 3-6-40 Momochihama, Sawara-ku, Fukuoka 814-0001, Japan

**Keywords:** Intranerve cross-sectional area variability, Nerve ultrasound, Nerve cross-sectional area, Chronic inflammatory demyelinating polyradiculoneuropathy, Multifocal motor neuropathy

## Abstract

**Objective:**

We aimed to evaluate differences in ultrasonographic nerve enlargement sites among typical chronic inflammatory demyelinating polyradiculoneuropathy (CIDP), distal CIDP, multifocal CIDP and multifocal motor neuropathy (MMN) in a Japanese population.

**Methods:**

We retrospectively reviewed medical records and selected 39 patients (14 with typical CIDP, 7 with multifocal CIDP, 4 with distal CIDP, and 14 with MMN) who underwent ultrasonography. Median and ulnar nerve cross-sectional areas (CSAs) were measured at the wrist, forearm, elbow, and upper arm. CSA ratios for each nerve were calculated as: wrist-to-forearm index (WFI) = wrist CSA/forearm CSA; elbow-to-upper arm index (EUI) = elbow CSA/upper arm CSA; and intranerve CSA variability (INCV) = maximal CSA/minimal CSA.

**Results:**

Significant differences were observed among typical CIDP, multifocal CIDP, distal CIDP, and MMN in CSA at the forearm and upper arm in the median nerves (p < 0.05). Patients with multifocal CIDP had lower WFI and EUI and higher INCV than the other groups (p < 0.05).

**Conclusions:**

Regardless of the untreated period, compared with other CIDP subtypes and MMN, multifocal CIDP showed a focal and marked nerve enlargement in the Japanese population.

**Significance:**

Differences in nerve enlargement site may be an underlying feature of multifocal CIDP.

## Highlights

• Sites of nerve enlargement on ultrasonography differ among typical CIDP, CIDP variants, and MMN.
• Multifocal CIDP showed focal and marked nerve enlargement compared with other CIDP variants and MMN.
• Differences in nerve enlargement sites may be an underlying feature of multifocal CIDP.

## 1 Introduction

Chronic inflammatory demyelinating polyradiculoneuropathy (CIDP) is an autoimmune disorder classified into six types based on clinical and electrophysiological features: typical, distal, multifocal, focal, motor, and sensory (Van den Bergh et al., 2021). Multifocal motor neuropathy (MMN), also an autoimmune peripheral neuropathy, is an asymmetric pure motor neuropathy with multifocal conduction blocks (Joint Task Force of the EFNS and the PNS, 2010).

In these diseases, ultrasonography (US) has been established as a supportive tool and its use can help diagnose CIDP in patients fulfilling the diagnostic criteria for possible CIDP according to the revised guidelines (Van den Bergh et al., 2021). However, few studies have reported the differences in US findings between different CIDP variants and MMN (Kerasnoudis et al., 2015, Kerasnoudis et al., 2016, Grimm et al., 2016). Moreover, whether the location of nerve enlargement differs among different subtypes remains unclear. Therefore, we evaluated the cross-sectional area (CSA) as well as the intranerve cross-sectional area variability (INCV) (Padua et al., 2012), which are CSA ratios within the same nerve. Additionally, we evaluated other CSA ratios, including the wrist-to-forearm index (WFI) and elbow-to-upper arm index (EUI).

This study aimed to assess the US pattern in the Japanese population using the CSA and associated ratios to determine their differences in patients with typical CIDP, multifocal CIDP, distal CIDP and MMN.

## 2 Methods

### 2.1 Study population

We retrospectively reviewed the medical records and selected patients diagnosed with CIDP or MMN who underwent US at our institution between January 2015 and February 2023. CIDP and its variants were diagnosed using the 2021 European Academy of Neurology/Peripheral Nerve Society guideline criteria (Van den Bergh et al., 2021), and MMN was diagnosed using the 2010 European Federation of Neurological Societies/Peripheral Nerve Society guideline criteria (Joint Task Force of the EFNS and the PNS, 2010). The condition was diagnosed by collecting available clinical symptoms, treatment status, history, blood test data, and electrophysiological findings from medical records. Data on the patient’s age, height, weight, and disease treatment duration at the time of US were extracted. The untreated period was calculated by subtracting treatment duration from disease duration.

This study was reviewed and approved by the Ethics Committee of our institution (Approval No. B210295). The requirement for written informed consent was waived because of the study’s retrospective design, and consent was obtained through an opt-out form. This form was made available on the internet; it detailed the outline of the research, purpose and methods, and use of materials, and included a point of contact to allow patients to refuse participation at any time.

### 2.2 US examination

Nerve US was performed using a 4–12 and 8–18 MHz linear array transducer (LOGIQ e; GE Healthcare Japan, Tokyo, Japan) and a 5–18 MHz linear array transducer (ARIETTA; Hitachi ALOKA Medical, Tokyo, Japan) by an examiner (Y.N.) with more than 10 years of experience in neuromuscular US. The frequency was set to 16 MHz at the median and ulnar nerves with LOGIQ e nerve preset and 18 MHz with ARIETTA, and 12 MHz at the cervical root with both LOGIQ e and ARIETTA MSK presets. The depth, focus, and zoom functions were standardized across all study examinations within and between patients. The axial images of the peripheral nerves were obtained, and the CSA was measured by tracing the nerve just inside the epineurium.

First, the entire nerve was scanned, and CSA of the median and ulnar nerves was measured at the wrist (in the median nerve, carpal tunnel inlet at the pisiform bone level; in the ulnar nerve, at the head of ulnar bone level); at the forearm (in the median and ulnar nerve, at the middle of forearm level); at the elbow (in the median nerve, at the antecubital fossa level; in the ulnar nerve, at the medial epicondyle level); and at the upper arm (in the median and the ulnar nerves; at the mid-point of the upper arm level). Additionally, CSA was measured for C5, C6, and C7 nerve roots at their exit point. US examination was performed on the symptomatic side; if performed bilaterally, the side with more prominent CSA enlargement was considered. The WFI, EUI, and INCV were calculated as follows (all CSAs measured within the same nerve):

$$\mathrm{WFI}=\frac{\mathrm{wrist}\;CSA}{\mathrm{forearm}\;CSA}$$

$$\mathrm{EUI}=\frac{\mathrm{elbow}\;CSA}{\mathrm{upper}\;arm\;CSA}$$

$$\mathrm{INCV}=\frac{\mathrm{maximal}\;CSA}{\mathrm{minimal}\;CSA}$$

### 2.3 Statistical analyses

Statistical analyses were performed using GraphPad Prism Version 8.41 for Windows (GraphPad Software, San Diego, CA). Categorical data were expressed as n (%), normally distributed variables as mean (standard deviation), and non-normally distributed variables as median (range). Fisher’s exact test was used for categorical data, analysis of variance for normally distributed variables, and the Kruskal–Wallis test for non-normally distributed variables. For *p*-values less than 0.05, Dunn’s test was performed to compare multifocal CIDP with the other groups. Correlations between CSA or ratios (WFI, EUI, INCV), and untreated period in each group were analyzed using Spearman’s rank correlation coefficient. However, in cases of ulnar nerve and cervical root CSA, correlation between the untreated period and CSA not analyzed because some groups had highly deficient CSA values. All significance tests were two-tailed, with p < 0.05 considered significant.

## 3 Results

A total of 39 patients (14 with typical CIDP, 7 with multifocal CIDP, 4 with distal CIDP, and 14 with MMN) were included.

### 3.1 Patient demographics

The demographic data of the study participants are shown in Table 1. Age, symptom onset age, disease duration, and untreated period differed significantly; however, no significant differences were found in sex, treatment duration, height, weight, or US device between the four groups.

**Table 1:** Demographic data of the study participants.

Variables	Typical CIDP (n = 14)	Multifocal CIDP (n = 7)	Distal CIDP (n = 4)	MMN (n = 14)	p
Age, years, mean (SD)	60.1 (11.0)	46.7 (14.1)	75.8 (12.6)	54.2 (11.2)	**<0.05**
Sex, male, n (%)	6 (42.9)	1 (14.3)	2 (50.0)	9 (64.3)	0.19
Onset age, years, mean (SD)	54.1 (13.5)	29.6 (14.6)	70.8 (11.6)	43.7 (7.9)	**<0.05**
Disease duration, years, median (range)	6.0 (0.33–22.0)	14.0 (6.5–37.0)	4.3 (3.5–8.8)	8.1 (2.0–32.0)	**<0.05**
Treated disease duration, years, median (range)	2.6 (0.00–22.0)	6.7 (0.00–28.0)	0.00 (0.00–4.0)	4.9 (0.00–11.0)	**0.26**
Untreated period, years, median (range)	0.50 (0.00–7.0)	4.0 (0.00–37.0)	3.7 (0.7–8.8)	2.5 (0.00–22.5)	<0.05
Height, cm, mean (SD)	163.4 (10.7)	163.0 (8.1)	157.1 (6.2)	166.8 (11.0)	0.48
Weight, kg, mean (SD)	58.7 (11.0)	57.7 (9.7)	53.9 (11.1)	59.0 (15.3)	0.93
Ultrasonography device, LOGIQ e, n (%)	11 (78.6)	4 (57.1)	3 (75.0)	9 (64.3)	0.73

p-values obtained using Fisher’s exact test, analysis of variance, or the Kruskal–Wallis test. p < 0.05 is shown in bold.

Abbreviations: CIDP, chronic inflammatory demyelinating polyradiculoneuropathy; MMN, multifocal motor neuropathy; SD, standard deviation.

### 3.2 Comparison of CSAs

**Fig. 1:**
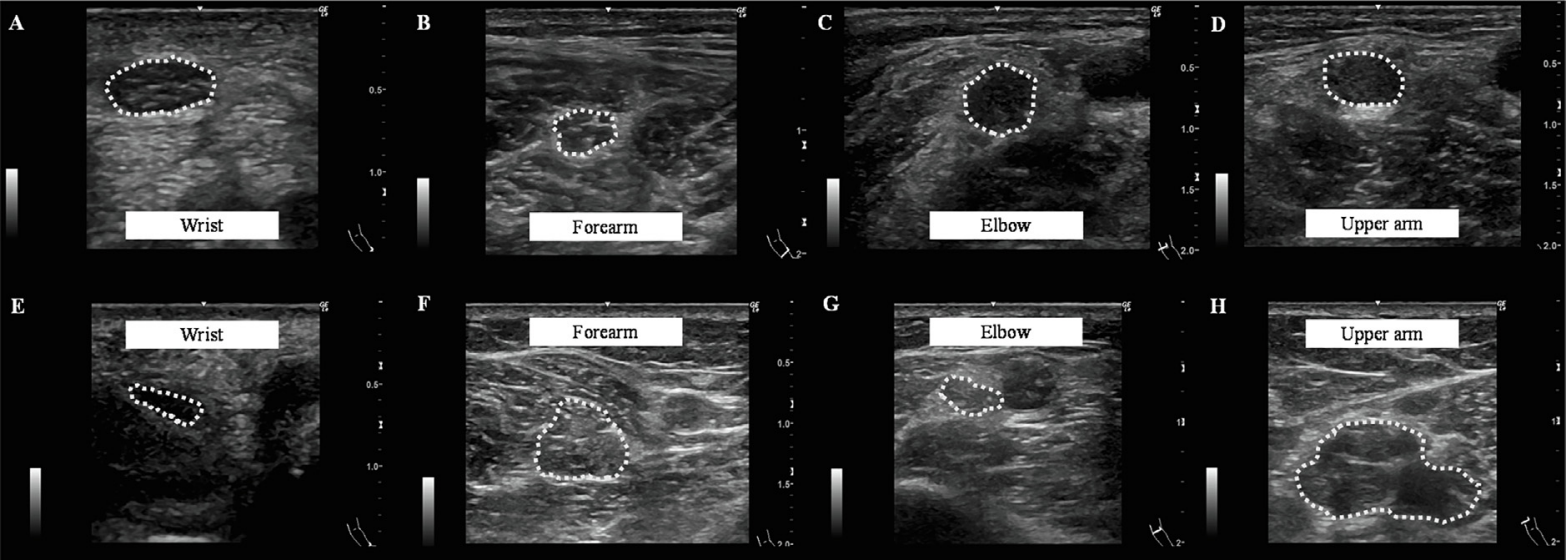
Representative ultrasound findings of the median nerve in a 65-year-old man with typical chronic inflammatory demyelinating polyneuropathy (CIDP) (A–D) and in a 53-year-old woman with multifocal CIDP (E–H). Each image shows the cross-sectional area (CSA) at the wrist (A = 18 mm^2^, E = 9 mm^2^), forearm (B = 16 mm^2^, F = 33 mm^2^), elbow (C = 26 mm^2^, G = 11 mm^2^), and upper arm (D = 21 mm^2^, H = 55 mm^2^). The wrist-to-forearm index (WFI) is 1.13 (typical CIDP) vs. 0.27 (multifocal CIDP); elbow-to-upper arm index (EUI) is 1.24 (typical CIDP) vs. 0.20 (multifocal CIDP); intranerve CSA variability (INCV) is 1.63 (typical CIDP) vs. 6.11 (multifocal CIDP).

**Fig. 2:**
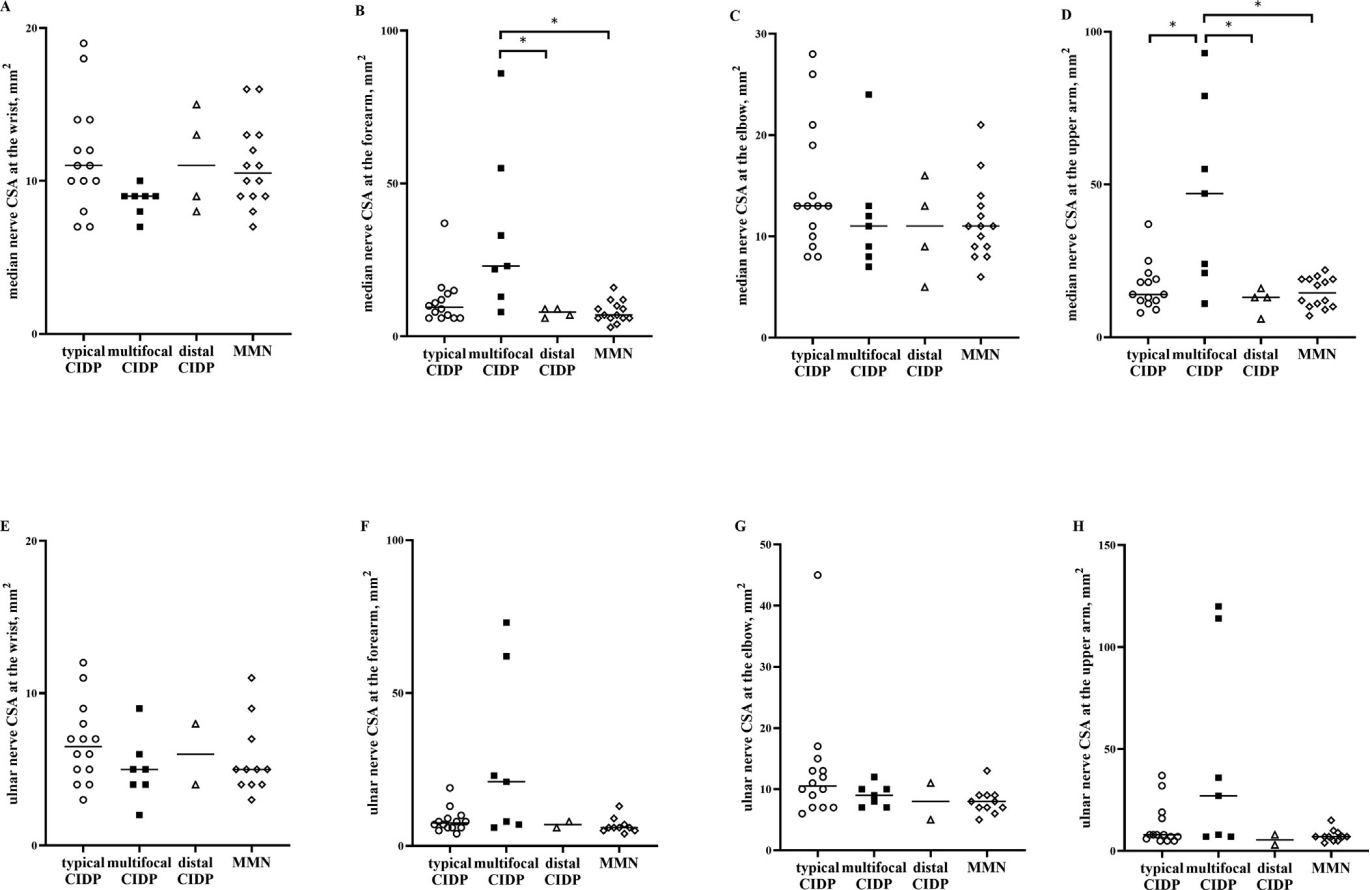
Comparison of cross-sectional area (CSA) in the median nerve and ulnar nerve at various sites among disease subtypes. A-D: median nerve CSA, E-H: ulnar nerve CSA. A, E: CSA at the wrist, B, F: CSA at the forearm, C, G: CSA at the elbow, D, H: CSA at the upper arm. White circle: typical chronic inflammatory demyelinating polyradiculoneuropathy (CIDP); black square: multifocal CIDP; white triangle: distal CIDP; white diamond: multifocal motor neuropathy (MMN). * p < 0.05 with post-hoc analysis.

**Fig. 3:**
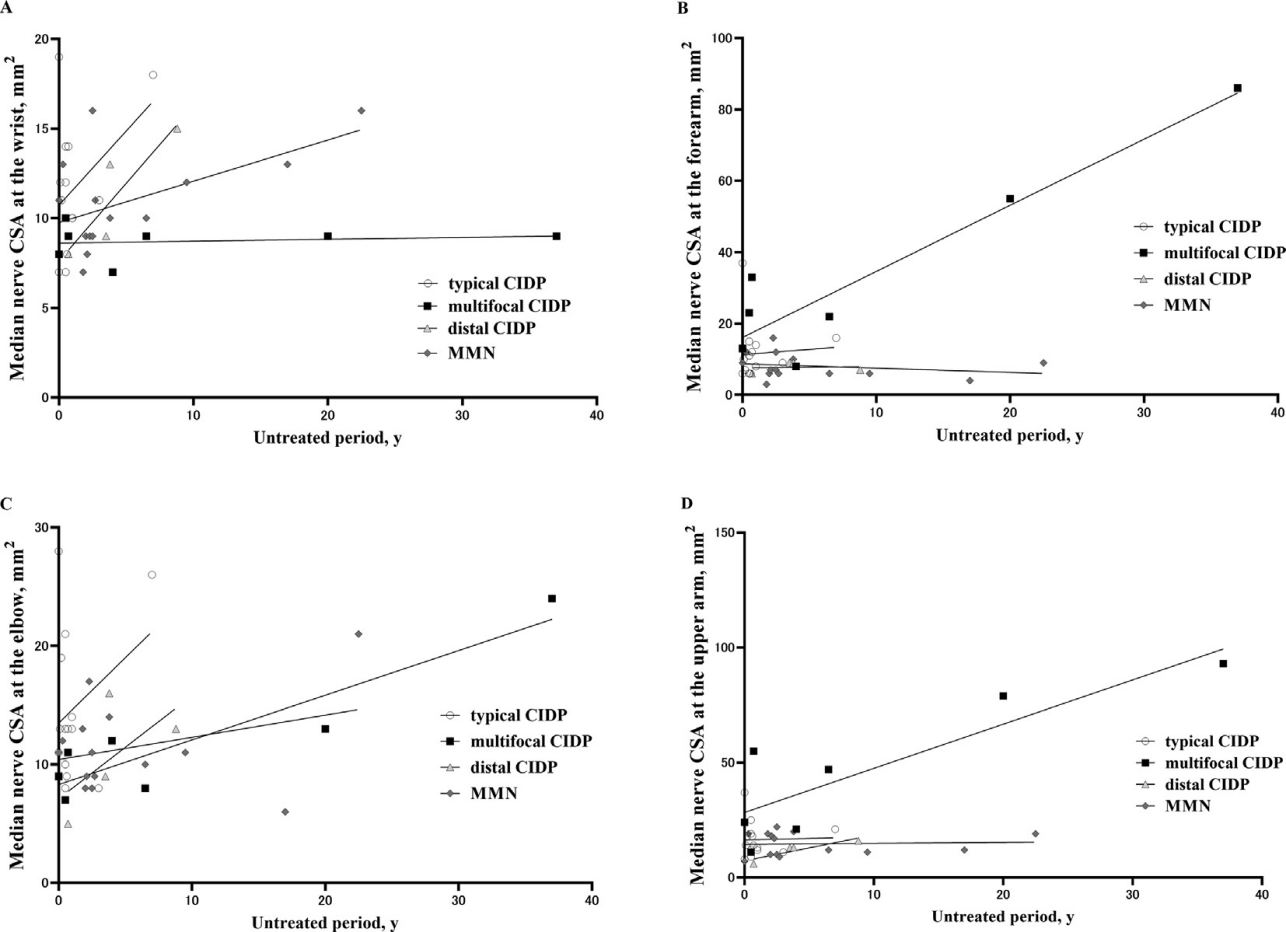
Relationship between median nerve CSA at each site and untreated period length. A: CSA at the wrist, B: CSA at the forearm, C: CSA at the elbow, D: CSA at the upper arm. White circle: typical chronic inflammatory demyelinating polyradiculoneuropathy (CIDP), black square: multifocal CIDP, gray triangle: distal CIDP, dark gray diamond: multifocal motor neuropathy (MMN). Each regression line is shown as a black line.

Representative US findings of the median nerve in patients with typical and multifocal CIDP are shown in Fig. 1. The median nerve CSA in the forearm and upper arm was larger in multifocal CIDP than in typical CIDP. Both WFI (0.27 vs. 1.13) and EUI (0.20 vs. 1.24) were lower in multifocal CIDP, and INCV (6.11 vs. 1.63) was higher in multifocal CIDP than in typical CIDP. CSAs of the median nerve and ulnar nerve at each site in all disease subtypes are shown in Fig. 2. There were significant differences among the four groups in CSA at the forearm and upper arm in the median nerves (p < 0.05, Kruskal–Wallis test). The median nerve CSA at the forearm in multifocal CIDP was significantly larger compared with distal CIDP and MMN according to post-hoc analysis (p < 0.05, post-hoc Dunn’s multiple comparison). The median nerve CSA at the upper arm in multifocal CIDP was significantly larger compared with typical CIDP, distal CIDP, and MMN according to post-hoc analysis (p < 0.05, post-hoc Dunn’s multiple comparison). The ulnar nerve CSA and cervical root CSA were not significantly different at any site (p > 0.05, Kruskal–Wallis test). All CSA measurements are shown in Table 2. The relationship between the median nerve CSA at all sites and the untreated period is shown in Fig. 3. No correlation was observed for any disease group (p > 0.05, Spearman’s rank correlation coefficient).

**Table 2:** Comparison of the CSAs.

Variables	Typical CIDP (n = 14)	Multifocal CIDP (n = 7)	Distal CIDP (n = 4)	MMN (n = 14)	p
**Median nerve CSA, mm^2^, median (range)**					
– Wrist	11.0 (7.0–19.0)	9.0 (7.0–10.0)	11.0 (8.0–15.0)	10.5 (7.0–16.0)	0.15
– Forearm	9.5 (6.0–37.0)	23.0 (8.0–86.0)	8.0 (6.0–9.0)	7.0 (3.0–16.0)	**<0.05**
– Elbow	13.0 (8.0–28.0)	11.0 (7.0–24.0)	11.0 (5.0–16.0)	11.0 (6.0–21.0)	0.43
– Upper arm	14.0 (8.0–37.0)	47.0 (11.0–93.0)	13.0 (6.0–16.0)	14.5 (7.0–22.0)	**<0.05**
*Ulnar nerve CSA, mm^2^, median (range)					
– Wrist	6.5 (3.0–12.0)	5.0 (2.0–9.0)	*6.0 (4.0–8.0)	*5.0 (3.0–11.0)	0.46
– Forearm	7.5 (4.0–19.0)	21.0 (6.0–73.0)	*7.0 (6.0–8.0)	*6.0 (4.0–13.0)	0.05
– Elbow	10.5 (6.0–45.0)	9.0 (7.0–12.0)	*8.0 (5.0–11.0)	*8.0 (5.0–13,0)	0.16
– Upper arm	8.0 (5.0–37.0)	27.0 (7.0–120.0)	*5.5 (3.0–8.0)	*7.0 (4.0–15.0)	0.1
*Cervical root CSA, mm^2^, median (range)					
– C5	*11.0 (4.0–16.0)	6.0 (5.0–12.0)	*9.0 (5.0–14.0)	*8.5 (4.0–13.0)	0.40
– C6	15.5 (3.0–34.0)	13.0 (7.0–37.0)	14.5 (5.0–32.0)	*9.0 (4.0–23.0)	0.47
– C7	*15.0 (5.0–33.0)	*13.0 (5.0–37.0)	*13.0 (8.0–18.0)	*11.0 (10.0–27.0)	0.48

p-values obtained using the Kruskal–Wallis test. p < 0.05 is shown in bold.

Abbreviations: CIDP, chronic inflammatory demyelinating polyradiculoneuropathy; MMN, multifocal motor neuropathy; CSA, cross-sectional area.

* Deficit data; all sites in the ulnar nerve, n = 2 in distal CIDP; wrist, elbow, and upper arm in ulnar nerve, n = 3 in MMN; forearm in ulnar nerve, n = 4 in MMN; C5, n = 2 in typical CIDP, n = 1 in distal CIDP, n = 6 in MMN; C6, n = 3 in MMN; C7, n = 1 in typical CIDP, n = 1 in multifocal CIDP, n = 2 in distal CIDP, n = 4 in MMN.

### 3.3 Comparison of WFI, EUI, and INCV

**Fig. 4:**
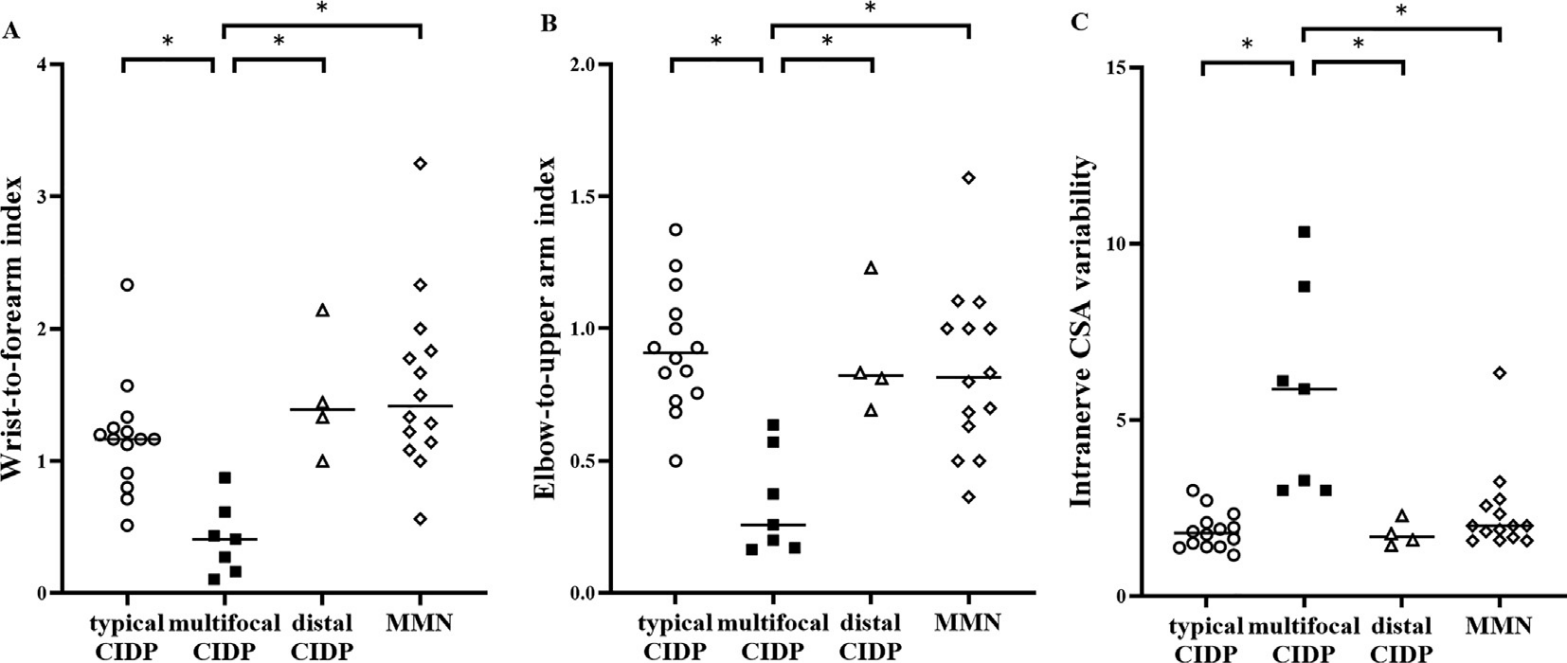
Wrist-to-forearm index **(**WFI), elbow-to-upper arm index (EUI), and intranerve CSA variability (INCV) in median nerve among disease subtypes. A: WFI in median nerve, B: EUI in median nerve, C: INCV in median nerve. White circle: typical chronic inflammatory demyelinating polyradiculoneuropathy (CIDP); black square: multifocal CIDP; white triangle: distal CIDP; white diamond: multifocal motor neuropathy (MMN). *p < 0.05 with post-hoc analysis.

**Fig. 5:**
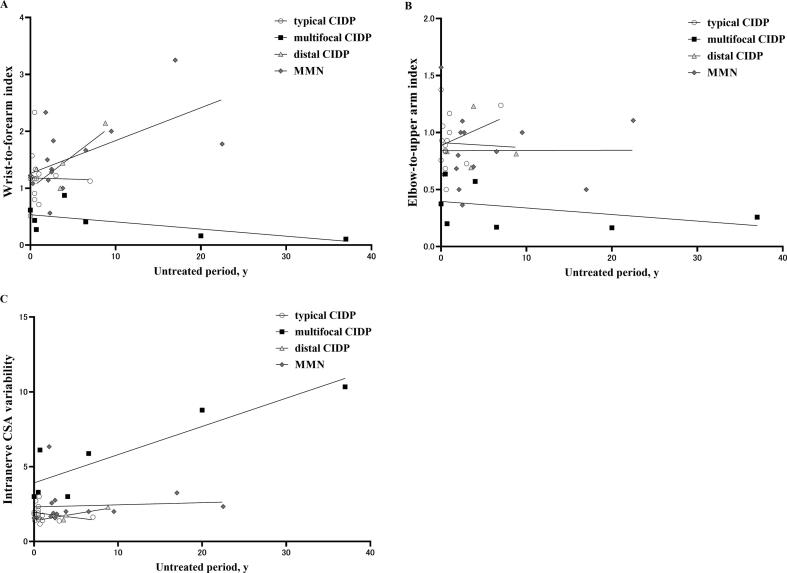
Relationship between the median nerve wrist-to-forearm index (WFI), elbow-to-upper arm index (EUI), or intranerve CSA variability (INCV) and untreated period length. A: WFI in median nerve, B: EUI in median nerve, C: INCV in median nerve. White circle: typical chronic inflammatory demyelinating polyradiculoneuropathy (CIDP); black square: multifocal CIDP; gray triangle: distal CIDP; dark-gray diamond: multifocal motor neuropathy (MMN). Each regression line is shown as a black line**.**

In the median nerve, there were significant inter-group differences in WFI, EUI, and INCV (p < 0.05 in all subtypes, Kruskal–Wallis test). Multifocal CIDP had a lower WFI and EUI and higher INCV compared with typical CIDP, distal CIDP, and MMN according to the post-hoc analysis (p < 0.05, post-hoc Dunn’s multiple comparison, Fig. 4). In the ulnar nerve, although missing values led to lowered statistical reliability, there were significant inter-group differences in WFI and EUI (p < 0.05 in both cases, Kruskal–Wallis test). Multifocal CIDP had a lower WFI compared with typical CIDP and MMN (p < 0.05 in both cases, post-hoc Dunn’s multiple comparison) and a lower EUI compared with typical CIDP and distal CIDP (p < 0.05 in both cases, post-hoc Dunn’s multiple comparison). The relationship between the median nerve WFI, EUI, or INCV and the untreated period is shown in Fig. 5. No correlation was observed for typical CIDP (WFI: r = 0.06, p = 0.84, EUI: r = 0.03, p = 0.92, INCV: r = −0.40, p = 0.15), multifocal CIDP (WFI: r = −0.71, p = 0.09, EUI: r = −0.57, p = 0.20, INCV: r = 0.77, p = 0.051), distal CIDP (WFI: r = 0.80, p = 0.33, EUI: r = 0.00, p > 0.99, INCV: r = 0.80, p = 0.33), or MMN (WFI: r = 0.45, p = 0.11, EUI: r = 0.09, p = 0.76, INCV: r = 0.39, p = 0.17). Note that correlations were not analyzed in the ulnar nerve because of missing CSA values, which reduced statistical reliability. All WFI, EUI, and INCV measurements are shown in Table 3.

**Table 3:** Comparison of the WFI, EUI, and INCV.

**Variables**	**typical CIDP (n = 14)**	**multifocal CIDP (n = 7)**	distal CIDP (*n = 4)	MMN (*n = 14)	**p**
**Median nerve index, median (range)**					
– WFI	1.17 (0.51–2.33)	0.41 (0.10–0.88)	1.39 (1.00–2.14)	1.42 (0.56–3.25)	**<0.05**
– EUI	0.91 (0.50–1.38)	0.26 (0.16–0.64)	0.82 (0.69–1.23)	0.82 (0.36–1.57)	**<0.05**
– INCV	1.79 (1.17–3.00)	5.88 (3.00–10.33)	1.69 (1.44–2.29)	2.00 (1.57–6.33)	**<0.05**
*Ulnar nerve index, median (range)					
– WFI	0.79 (0.57–1.50)	0.33 (0.05–0.75)	*0.83 (0.67–1.00)	*0.83 (0.60–1.25)	**<0.05**
– EUI	1.21 (0.38–3.00)	0.44 (0.08–1.00)	*1.52 (1.38–1.67)	*1.14 (0.53–1.86)	**<0.05**
– INCV	2.33 (1.33–4.09)	3.50 (1.33–30.00)	*1.69 (1.38–2.00)	*2.00 (1.40–2.60)	0.21

p-values obtained using the Kruskal–Wallis test. p < 0.05 is shown in bold.

Abbreviations: CIDP, chronic inflammatory demyelinating polyradiculoneuropathy; MMN, multifocal motor neuropathy; INCV, intranerve cross-sectional area variability; WFI, wrist-to-forearm index; EUI, elbow-to-upper arm index.

* Deficit data; wrist to forearm index in ulnar nerve, n = 2 in distal CIDP, n = 4 in MMN; elbow to upper arm index in ulnar nerve, n = 2 in distal CIDP, n = 3 in MMN; intra-nerve variability in ulnar nerve, n = 2 in distal CIDP, n = 4 in MMN.

## 4 Discussion

Typical CIDP and CIDP variants are diagnosed based on clinical features but are also characterized by their own electrophysiology (Kuwabara et al., 2015, Lehmann et al., 2019). Recent diagnostic guidelines describe the usefulness of US in diagnosis; however, they do not mention the characteristics of nerve enlargement among CIDP variants and MMN (Van den Bergh et al., 2021). This study showed that nerve enlargement differed among typical CIDP, multifocal CIDP, distal CIDP, and MMN in the Japanese population. In addition, multifocal CIDP showed focal and marked nerve enlargement compared with typical CIDP, distal CIDP, and MMN. In previous reports (Grimm et al., 2016, Décard et al., 2018), including the INCV study by Padua et al. (2012), multifocal CIDP and MMN were found to have more localized nerve enlargement compared to typical CIDP, and our results showing focal nerve enlargement and high INCV in the median nerve in multifocal CIDP are roughly consistent with this.

Our study has two unique results. First, the multifocal CIDP group had more pronounced nerve enlargement at the forearm and upper arm, especially in the median nerve, and the WFI and EUI were lower. Our sample of patients with multifocal CIDP was small in number and had a prolonged non-treatment period compared with typical CIDP, distal CIDP, and MMN. In previous reports, the relationship between treatment and nerve enlargement in CIDP (including multifocal CIDP) and MMN was thought to be heterogeneous (Décard et al., 2018, Niu et al., 2019). According to the relationships between the median nerve CSA at all sites with the untreated periods in Fig. 3, we noticed that the median nerve CSA at proximal sites other than the wrist in multifocal CIDP tended to increase with the untreated period, although it was not significant due to the small number of cases. This may have influenced the calculated INCV ratio in multifocal CIDP in Fig. 5. In contrast, multifocal CIDP always showed a different calculated ratio, such as WFI, EUI and INCV, from other subtypes, which may be an underlying feature beyond the influence of untreated period length in Fig. 5.

The second unique aspect is that there were significant differences in INCV, WFI, and EUI between multifocal CIDP and MMN. In a previous report, MMN had more localized nerve enlargement than multifocal CIDP and rarely extended to the entire nerve, thus resulting in increased INCV (Padua et al., 2012, Décard et al., 2018). Some reports suggest that the background pathology differs between MMN and CIDP (Garg et al., 2019). In addition, nerve enlargement may be more headward as only the motor nerves are involved in MMN and the complications of nerve atrophy due to axonal injury. Thus, this result may also reflect differences in the background pathology of MMN and multifocal CIDP.

The cause of these results remains unclear. Electrophysiological profiles suggest that multifocal CIDP is more affected in the nerve trunk than in terminals (Kuwabara et al., 2015, Ikeda et al., 2019), and further pathological and magnetic resonance imaging studies indicate that proximal nerves are more susceptible to multifocal CIDP (Beecher et al., 2021). However, this is not a satisfactory explanation for the lack of nerve enlargement at the wrist and elbow. Further investigations are required to clarify the underlying mechanisms.

This study has some limitations. The study design was retrospective, with a small sample size. The US data were unilateral, and there was the possibility of selection bias. Patients with multifocal CIDP had a younger age of onset, were more often women, and a longer disease duration, while those with distal CIDP had an older age of onset, which may have influenced the results. Furthermore, some patients with multifocal CIDP had significant variance within their data, and these outliers might have affected the results. Contrastingly, such outliers may be characteristic of multifocal CIDP and thus were included in the final results. Finally, we are concerned that the CSA of the distal ulnar nerve is too small, and hence, the INCV tends to be large, considering the error of measuring a smaller CSA than it should be. We examined this point in detail, and in the present study, there was no tendency for the INCV to be excessively high in cases with low minimum CSA values compared to others. Therefore, we judged that the influence of this point on the results was small, but the results should be treated with caution because of the small number of cases. We hope that prospective, large-scale data will be collected in future studies to validate our findings.

## 5 Conclusions

Nerve enlargement sites on US were significantly different among typical CIDP, multifocal CIDP, distal CIDP, and MMN. Compared to typical CIDP, distal CIDP, and MMN, multifocal CIDP showed a focal and marked nerve enlargement in the Japanese population, regardless of untreated period length. This may be a characteristic morphological underlying feature of multifocal CIDP.

## Funding

This research did not receive any specific grant from funding agencies in the public, commercial, or not-for-profit sectors.

## CRediT authorship contribution statement

**Masaaki Yoshikawa:** Conceptualization, Data curation, Formal analysis, Methodology, Writing – review & editing, Investigation. **Kenji Sekiguchi:** Conceptualization, Supervision, Funding acquisition, Methodology, Investigation, Writing – review & editing, Formal analysis, Data curation. **Hirotomo Suehiro:** Data curation, Writing – review & editing, Investigation. **Shunsuke Watanabe:** Data curation, Writing – review & editing, Investigation. **Yoshikatsu Noda:** Conceptualization, Writing – review & editing, Investigation, Methodology, Data curation, Formal analysis. **Hideo Hara:** Supervision, Writing – review & editing. **Riki Matsumoto:** Conceptualization, Supervision, Writing – original draft, Writing – review & editing.

## Declaration of competing interest

The authors declare that they have no known competing financial interests or personal relationships that could have appeared to influence the work reported in this paper.
